# Screening tools for malignancy in patients with cryptogenic stroke: Systematic review

**DOI:** 10.1177/23969873241310760

**Published:** 2025-02-26

**Authors:** Maria P Tieck, Constanze Single, Sven Poli, Markus C Kowarik, Ulf Ziemann, Annerose Mengel, Katharina Feil

**Affiliations:** 1 Department of Neurology & Stroke, University Hospital Tübingen, Tübingen, Germany; 2 Hertie-Institute for clinical Brain Research, University Hospital Tübingen, Tübingen, Germany

**Keywords:** Occult malignancy, ischemic stroke of undetermined source, malignancy screening

## Abstract

Up to 20% of patients with cryptogenic ischemic stroke have an occult malignancy at the time of stroke presentation, providing an opportunity for early cancer detection. Despite this significant association, there is currently no consensus in international guidelines on how to systematically screen for malignancy in ischemic stroke patients. This review aims to summarize recent evidence on clinical features and scores, and predictive laboratory tests, that can guide malignancy screening in ischemic stroke patients. Our systemic search included PubMed, MEDLINE and Cochrane databases and yielded a total of 12 studies meeting the inclusion criteria for review. Elevated D-dimer levels and multiple infarcts in different cerebral circulations emerged as key markers. Based on the summarized data, we propose a flowchart for clinical decision-making regarding malignancy screening in patients with ischemic stroke. As the initial steps, we recommend using D-dimers cut-offs and stroke pattern on brain imaging to classify patients according to their risk profile. Based on the identified risk, we recommend a subsequent diagnostic workup addressing the most prevalent cancer types, including gastrointestinal tract, lung adenocarcinoma and gender-related cancer. The clinical implications of early malignancy screening and the need for evidence-based guidelines in cryptogenic stroke are discussed.

## Graphical abstract

**Graphical abstract:**
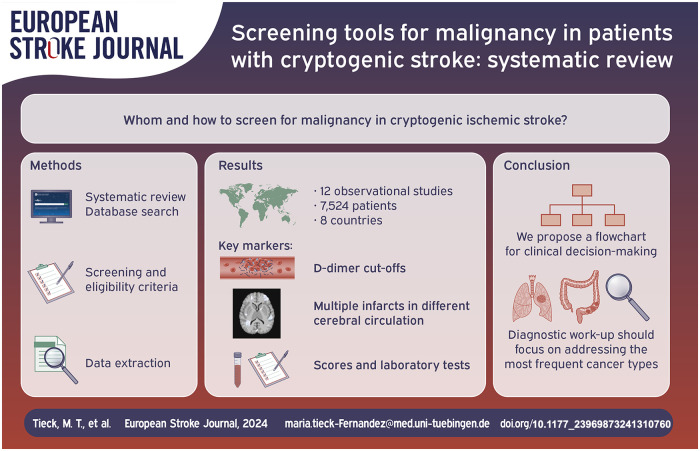

## Introduction

An acute ischemic stroke can be the first manifestation of an underlying malignancy. Cancer-related hypercoagulability may provoke thromboembolic cerebral events through paradoxical embolism of venous thrombosis, nonbacterial thrombotic endocarditis, atrial fibrillation, and atherosclerosis.^1^ The risk of occult malignancy appears highest in patients with cryptogenic ischemic stroke. Around 50% of cancer associated ischemic strokes are classified cryptogenic after the initial evaluation.^2^ Prevalence of malignancy among cryptogenic ischemic stroke patients reaches nearly 20%, significantly higher than the 6% observed in all-cause ischemic stroke patients.^3^ The median interval between stroke and cancer diagnosis in patients with occult malignancy ranges from 1 to 8 months in the majority of cases.^4^

Due to an innate hypercoagulable state caused by malignancy, the risk for venous and arterial thromboembolism is higher when compared to the general population.^5^ Thrombosis is enhanced due to the release of procoagulants such as factor X and inflammatory cytokines by tumor cells. Increased D-dimers and high levels of fibrinogen in these patients are associated with more thromboembolic events.^3^ Whereas venous thromboembolism is mostly due to the activation of the plasmatic clotting system, platelet activation is driving arterial thromboembolism. Although venous and arterial thromboembolism differ in their pathophysiology, malignancy related stroke patients have a higher risk for both, subsequent arterial, and venous thromboembolic events.^6^ Among arterial events, ischemic strokes are more common than other arterial thromboembolism. In a cumulative cohort of 279,719 cancer patients and matched controls the incidence of ischemic stroke was 3.0% versus 1.6% and of myocardial infarction 2.0% versus 0.7%.^5^ Venous thromboembolic events are however more common than arterial events with an incidence that varies between 4% and 20%.^7^

Solid tumors are the most common type of cancer in patients with malignancy related ischemic stroke. Around 60% of cancer associated with ischemic stroke are adenocarcinomas, 20%–30% squamous cell carcinomas and less than 10% sarcoma and lymphomas. However, the prevalence of different types of cancer varies between studies.^8^ Adenocarcinomas are of glandular origin and include prostate, breast, pancreatic, gastrointestinal, and lung cancer. The prevalence of cancers in ischemic stroke patients is similar to that in the general elderly population, with lung, colorectal, prostate, gastric, and breast cancers being the most common.^3,9^ However, lung, pancreatic and colorectal cancers in specially in advance stages seem to carry the highest stroke risk.^3,10,11^

In the last decades several studies have tried to characterize ischemic stroke patients in order to identify predictors for malignancy.^3^ Factors such as advanced age, presence of chronic renal failure, elevated D-dimers, history of previous cancer, and smoking have been described as risk factors for malignancy in ischemic stroke patients.^12,13^ However, there are no general recommendations in any international guideline of how and whom to screen for malignancy.

The aim of this systematic review is to screen the current literature for evidence on malignancy predictors among patients with cryptogenic ischemic stroke and to suggest strategies for clinicians on how to guide an appropriate evidence-based malignancy screening.

## Methods

**Figure 1.**
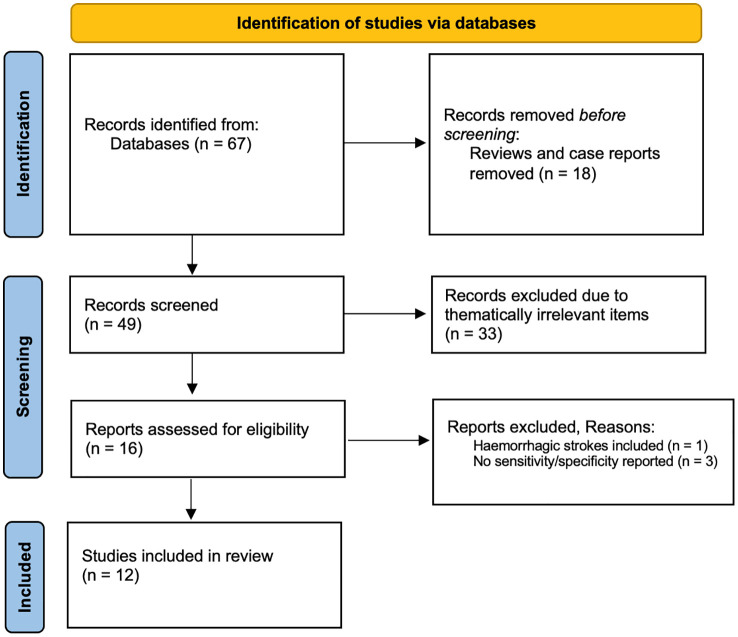
Flowchart of literature review process (PRISMA flowchart).

This systematic review followed the PRISMA guidelines (Supplementary PRISMA checklist).^14^ We performed a systematic search using PubMed, MEDLINE and Cochrane databases with the search query “(occult malignancy) AND ((embolic stroke of undetermined source) OR (cryptogenic stroke) OR (ischemic stroke) OR (embolic stroke))” without time restriction on August 15, 2024. Original research articles in English, German, or Spanish were considered if they reported sensitivity/specificity data obtained from studies on human ischemic stroke, but not hemorrhagic stroke. We excluded reviews and case reports (Figure 1).

### Study screening and selection

Two independent reviewers (MPT and CS) screened the titles, abstracts, and full texts. Conflicts were resolved by a third reviewer. The final selection included 12 studies based on inclusion/exclusion criteria, with reasons for exclusion documented. In addition, we examined papers cited in the selected articles and included additional references based on their relevance regarding the scope of this paper.

### Risk of bias assessment

The risk of bias was assessed using the Risk Of Bias In Non-randomized Studies (ROBINS) tool (see Supplemental Figure 1).^15^

## Results

### Included studies

The search resulted in 67 records after the removal of duplicates (Figure 1). After full-text eligibility assessment of 16 records, 12 were included. The publication year ranged from 2012 to 2022.

### Study characteristics

The 12 studies comprised 7524 patients. Half of the study population were from Asia, the other half being from Europe and the USA and used hospital-based samples. 11 studies were observational retrospective. Only 1 study was observational prospective. Nine out of the 12 (75%) studies included all stroke etiologies. Most of the studies were deemed at high risk of bias arising from measurement of the outcome (see Supplemental Figure 1). This was largely attributed to the individual decision to screen for malignancy, as well as the absence of a systematic diagnostic workup assessment.

### Markers

#### Involved cerebral circulations

Malignancy related ischemic strokes show mainly an embolic infarct pattern, and over 70% affect multiple cerebral circulations.^11^ Involvement of more than one cerebral circulation on diffusion-weighted magnetic resonance imaging has a high sensitivity of 92% but a rather low specificity of 65% for malignancy detection. With the involvement of all three cerebral circulations, which is known as the three-territory sign or Trousseau syndrome, the specificity increases to up to 96% for occult malignancy related stroke.^16^ A study comparing embolic infarct patterns between ischemic stroke patients with underlying atrial fibrillation and active malignancy demonstrated a three-territory involvement in 3.5% and 23.4% of cases, respectively.^17^

#### D-dimers

D-dimers have been deeply studied in hypercoagulability states including cancer. A variety of studies show an association of elevated D-dimer levels and occult malignancy among ischemic stroke patients. But it is not to forget that D-dimers are in general elevated also by cardioembolic stroke compared to other stroke etiologies.^18^ Nevertheless, D-dimer levels tend to be even higher in patients with occult malignancy related stroke.^17^ The malignancy associated coagulopathy greatly contributes to thromboembolic events in cancer patients. A prospective study of 74 ischemic stroke patients with active cancer showed that almost half of the patients had microembolic signals on transcranial Doppler ultrasound. The presence of microemboli was associated with high D-dimer levels, suggesting an active central embolic source and/or a hypercoagulable state.^19^ Several studies have addressed the question of a potential threshold of D-dimer levels alone or in combination with other markers including hemoglobin or magnetic resonance imaging findings that may trigger malignancy screening among ischemic stroke patients. For D-dimers, sensitivity and specificity varies among different studies and cut-off values applied. Sensitivity may vary from 31% to 89% and specificity from 65% to 99% depending on the used cut-off value. Table 1 describes D-Dimers in an ascending order and their respective sensitivity and specificity according to the identified studies.

**Table 1.** Diagnostic value of different markers and multiple scores for occult malignancy prediction in ischemic stroke patients.

Studies	Markers and scores	Scoring	Sensitivity (%)	Specificity (%)	PPV (%)	NPV (%)	Studied types of ischemic stroke population	Number of patients	Study design^a^	Country	Years of data collection
Guo et al.^10^	D-dimers > 0.55 mg/L	-	79.6	66.7	33.5	94.0	All etiologies	516	Retrospective	Taiwan	3
Rosenberg et al.^18^	D-dimers > 1.2 mg/L	-	85	65	-	-	All etiologies	480	Retrospective	USA	1,5
Guo et al.^10^	D-dimers > 1.55 mg/L	-	59.2	91.8	60.4	91.5	All etiologies	516	Retrospective	Taiwan	3
Kim et al.^11^	D-dimers > 2.15 mg/L	-	85.7	96.6	88.9	95.5	Cryptogenic etiology	348	Retrospective	Korea	5
Tsushima et al.^20^	D-dimers > 2.68 mg/L	-	89	72	-	-	All etiologies	496	Retrospective	Japan	3
	CRP > 0.29 mg/dL	-	80	63	-	-					
Guo et al.^10^	D-dimers > 5.5 mg/L	-	31.6	99.6	93.9	87.4	All etiologies	516	Retrospective	Taiwan	3
	D-dimers > 0.55 mg/L + multiple infarcts in multiple cerebral circulations	-	24	99.7	92.9	90					
	D-dimers > 1.55 mg/L + multiple infarcts in multiple cerebral circulations	-	16.7	100	100	88.9					
Selvik et al.^21^	D-dimers > 3 mg/L + Hb <12 g/dL + smoking history	-	21	99	-	-	All etiologies	1646	Retrospective	Norway	6
Jiang et al.^22^	- D-Dimer > 2.0 mg/L	1/3	96	37	-	-	All etiologies	799	Retrospective	China	1,5
	- Fibrinogen > 400 mg/dL	2/3	68	88	-	-					
	- Absence of hyperlipidemia	3/3	19	99	-	-					
Liu et al.^23^	MOCHA profile - D-dimers > 0.5 mg/L - Prothrombin fragment 1.2 > 288 pmol/L - Thrombin anti-thrombin complex > 5.5 µg/L - Fibrin monomer > 7 µg/mL	>2 abnormal markers	96	62	23	99	Cryptogenic etiology	236	Prospective	USA	3
Beyeler et al.^8^	OCCULT-5 score	⩾3	63.6	72.9	5	98.8	All etiologies	1001	Retrospective	Switzerland	1,5
	- Age of 77 years or older	4	27.2	91	6.4	98.2					
	- ESUS	5	4.5	98.7	7.6	97.8					
	- Multiple infarcts in multiple cerebral circulations - D-dimers > 0.8 mg/L - Female sex										
Nouh et al.^17^	Infarcts in all three cerebral circulations	-	23.4	96.4	-	-	Malignancy-related stroke and atrial fibrillation-related stroke	231	Retrospective	USA	2
Guo et al.^16^	Infarcts in more than one cerebral circulation	-	92	65	-	-	Cryptogenic atiology	108	Retrospective	China	6
Cocho et al.^24^	Fibrinogen > 600 mg/dL		67	91	-	-	All etiologies	631	Retrospective	Spain	3
			75	96							

a All Studies were observational studies.

NPV: negative preditctive value; PPV: positive predictive value.

#### Other blood biomarkers

Malignancy carries a systemic inflammatory state mostly due to cytokines released by tumor cells, therefore inflammatory markers are in general increased in these patients.^25^ Markers such as C-reactive protein (CRP) and fibrinogen in certain ranges have shown indirect evidence of occult malignancy. A retrospective study of 631 patients with ischemic stroke showed that a CRP above 2.0 mg/dL predicted malignancy with a sensitivity of 75% and a specificity of 96%, and fibrinogen levels above 600 mg/dL with a sensitivity of 67% and a specificity of 91%.^24^ A further study, comprising 486 ischemic stroke patients, revealed that CRP levels exceeding 0.29 mg/dL, which typically fall within the normal range, demonstrated a sensitivity of 80% and a specificity of 63% for the detection of malignancy upon initial admission to the hospital.^20^ Nevertheless, CRP must be carefully interpreted especially when attributing an increase to an occult malignancy. For instance, CRP is also increased in any other type of aseptic inflammation, is a marker of generalized atherosclerosis and it might be as well increased in cardioembolic stroke.^26,27^ Furthermore, CRP can be an indicator of an infectious process.^28^ In the case of infections, however, the elevation of CRP is usually accompanied with other markers such as leukocytes, procalcitonin and also clinical signs of infection such as fever.

### Taking all together – Scores and combined markers

Currently, there is no consensus on which ischemic stroke patients should undergo an extensive diagnostic workup for malignancy screening. Some authors have tried to combine markers to develop risk scores for prediction of occult malignancy (Table 1).

#### D-dimers, fibrinogen and absence of hyperlipidemia

A retrospective study of 799 stroke patients of all etiologies including cancer related ischemic stroke identified certain biomarkers in combination with clinical features to have a special predictive value for malignancy. Hemoglobin, platelet count, fibrinogen, D-dimers and CRP as well as demographic and clinical characteristics were compared between patients with ischemic stroke and active cancer versus those without known cancer. A clinical score was developed based on the absence of hyperlipidemia in the clinical records, serum fibrinogen levels of more than 4 g/L and D-dimer levels above 2 mg/L to predict active cancer among patients with cryptogenic ischemic stroke (see Table 1).^22^

#### D-dimers and multiple infarcts in multiple cerebral circulations

An embolic infarct pattern together with elevated D-dimers may increase probability to detect occult malignancy in ischemic stroke patients.^16^ D-dimers of 0.55 mg/L or more combined with multiple infarcts in multiple cerebral circulations showed near 100% specificity and a positive predictive value of 93% for cancer-related ischemic stroke.^10^ In a study of 348 cryptogenic ischemic stroke patients, all patients with an increased D-dimer level and an embolic infarct pattern on brain imaging had an occult cancer.^11^

#### Markers of Coagulation and Hemostatic Activation (MOCHA) profile

The MOCHA profile score uses different coagulation markers to predict occult malignancy in ischemic stroke patients and was evaluated in a prospective study. The markers included were D-dimers, prothrombin fragment 1.2, thrombin anti-thrombin complex, and fibrin monomer. One point is scored for each marker above normal.^23^ In this study, 13% of patients with a score of 2 or higher were diagnosed with malignancy compared to 1% of patients with a normal score. Moreover 5% of these were diagnosed with venous thromboembolism versus 0% of patients with a normal score.

#### OCCULT-5 score

A recent study of 1001 ischemic stroke patients of all etiologies retrospectively compared clinical and laboratory features in patients without malignancy to those of patients in whom malignancy was known at the time of stroke onset or newly diagnosed within the first year after stroke. Five markers and/or their features were found to be associated with malignancy and therefore included into the OCCULT-5 malignancy prediction score, that is, age of 77 years or older, embolic stroke of undetermined source, multiple infarcts in multiple cerebral circulations, D-dimer level >0.8 mg/L, and female sex. A reasonable clinical relevance was achieved with a score of 3 or more points (see Table 1).^8^

### Diagnostic workup and flowchart for detecting malignancy in cryptogenic ischemic stroke

**Figure 2.**
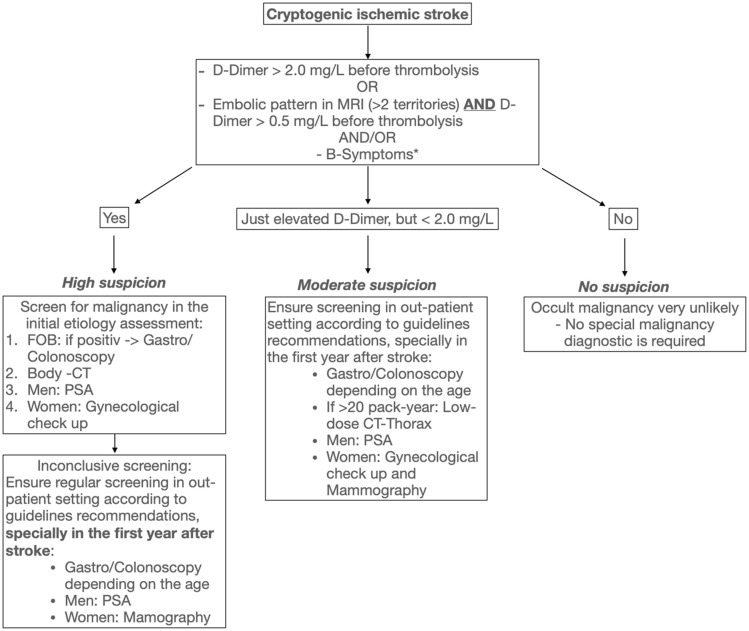
Flowchart for clinical decision-making in regard to malignancy screening in patients with cryptogenic ischemic stroke. FOB: fecal occult blood, PSA: prostate specific antigen. *In any case, if the clinician suspects malignancy due to the presence of other signs or symptoms, such as classic B-symptoms, a further workup is indicated at the clinician’s discretion

Based on the summarized evidence, we propose a flowchart to guide clinical decision making in screening cryptogenic ischemic stroke patients for occult malignancy (Figure 2). The presented flowchart was constructed based on the findings of the literature review. Although the identified studies were heterogeneous and most of them included ischemic stroke patients of all etiologies, our proposed flowchart is focusing on cryptogenic ischemic stroke patients, in whom an occult malignancy seems more probable. However, if an ischemic stroke with an apparent determined etiology (e.g. cardioembolism) reveals a strong D-dimer elevation (e.g. above 2 mg/L), these patients should also be considered for screening for malignancy. Classical sings like B symptoms, such as unintended weight loss, night sweats, and unexplained fever, may suggest malignancy; however, they are unspecific because they are also present in systemic autoimmune disorders, and other conditions.^29,30^ In the context of ischemic stroke, there is no specific current data linking B-symptoms and occult malignancy. If such symptoms are present in a cryptogenic ischemic stroke patient a further workup is indicated at the clinician’s discretion regardless of the presence or absence of the aforementioned markers.

The screening we propose is specially directed to the most common cancer types related to ischemic stroke which are adenocarcinomas of the lung, pancreas, gastrointestinal tract, breast, and prostate.^3^ In the following, we discuss the use and limitations of the most relevant diagnostic methods.

#### Body computed tomography

Computed tomography (CT) has shown to be a reliable tool for screening in certain cancer types, such as lung cancer.^31^ In the context of ischemic stroke, a body CT is frequently performed in cryptogenic etiology for malignancy screening.^18^ However, standard whole-body CT in all patients with cryptogenic ischemic stroke does not appear to be cost-effective, and radiation exposure needs to be considered. High sensitivity of body CT for malignancy detection was only shown in case of elevated D-dimers (see Table 1) which suggests limited utility of body CT if D-dimer levels are below the upper limit of normal.^18^ Cancer of the gastrointestinal tract is one of the most prevalent cancer types among stroke patients. However, computed tomography (CT) is insufficient for detecting pancreatic, gastric or colorectal cancer in its initial stages.^32^ Furthermore, the sensitivity of computed tomography for the detection of breast cancer is unknown,^33^ so it is fundamental to conduct focused screening with an adequate method to address these additional cancer types.

#### Fecal occult blood test and colorectal cancer

Fecal occult blood (FOB) test is a cheap and non-invasive diagnostic tool that could be easily performed in any patient. A positive FOB test was found to be associated with an increased risk of ischemic stroke and all cause of mortality, and should prompt for a further workup including colonoscopy to search for colorectal cancer.^34^ Of 1714 retrospectively assessed ischemic stroke patients, 3% were diagnosed with occult malignancy. Importantly, all the patients diagnosed with gastrointestinal cancer had a positive FOB test.^35^ In case of a negative FOB test result and unexplained stroke etiology, we recommend to repeat FOB test regularly in an ambulant setting. If positive, a colonoscopy should be guaranteed. International guidelines support annual FOB screening for the general population, due to a sustained 33% reduction per year in colorectal cancer related mortality.^36^

#### Colonoscopy and gastroscopy

Regardless the FOB test result, a gastroscopy and/or colonoscopy should be performed in patients with high risk for gastric cancer which include gastric adenomas, pernicious anemia, gastric intestinal metaplasia by chronic gastritis, familial adenomatous polyposis, lynch syndrome, juvenile polyposis syndrome.^37^ Regarding gastric cancer at least a gastroscopy should be performed specially in high-risk patients with previous chronic gastritis, gastric atrophy, gastric intestinal metaplasia, or dysplasia.^38^ The endoscopic view of the upper gastrointestinal tract afforded by gastroscopy also allows the detection of esophageal cancer.^39^

#### Sex-specific screening

Prostate-specific antigen test is the most used biomarker for prostate cancer screening. Elevated prostate-specific antigen may precede clinical manifestation of prostate cancer by up to 5–10 years.^40^ The most common female-related types of cancer are breast, ovarian, cervical and endometrial cancers. Of these, breast cancer is the most prevalent. In the case of breast cancer, for example, mammography, and breast ultrasound are the recommended methods of screening.^41^

#### Other diagnostic recommendations

Abdominal ultrasound may have some diagnostic value for renal cancer detection, but has no value for detecting gastrointestinal cancer and is therefore not suggested in any guideline.^42^ The gold standard for lung cancer screening is a low-dose lung CT, especially in patients with a >20 pack-year history of smoking.^43^ Screening for lung cancer with chest radiography is not sufficient and not recommended by any international guideline.^43^

Certain groups of patients who are at an exceptionally high risk for a specific cancer type should also undergo screening for occult malignancy. Pancreatic cancer screening should be considered in patients with genetic syndromes associated with an increased risk, including Peutz-Jeghers syndrome, hereditary pancreatitis, Cyclin Dependent Kinase Inhibitor 2A (CDKN2A) gene mutation, Lynch syndrome, and mutations in breast cancer 1 (BRCA1), BRCA2, partner and localizer of BRCA2 (PALB2), and ataxia-telangiectasia mutated (ATM) genes. MR cholangiopancreatography and/or endoscopic ultrasonography are the gold standard methods for screening.^44^

## Discussion

D-dimers and embolic infarct pattern on brain imaging are helpful tools to guide the clinician in selecting patients with cryptogenic ischemic stroke in whom to perform a malignancy screening. A number of clinical features have been identified as being associated with the diagnosis of occult malignancy in ischemic stroke patients in previous studies. These include an older age, a history of smoking, chronic obstructive pulmonary disease, congestive heart failure, chronic kidney disease, atrial fibrillation, previous non-active cancer.^45^ However, the sensitivity and specificity of these features alone have not yet been reported. D-dimers currently represent the most extensively investigated biomarker with consistent findings across several studies, and therefore the major focus of our study. D-dimers can be increased for a number of reasons, including surgery or trauma, pregnancy, infection, chronic inflammation, liver and renal disease, advanced age, and also transiently after thrombolytic therapy which is important to know in the context of ischemic stroke.^46^ Consequently, D-dimers must always be interpreted with caution. D-dimers alone yield high diagnostic value when using specific high cut-offs such as >2.15 mg/L.^11^ An unspecific less important D-Dimer elevation might be useful to detect high-risk patients, but only in combination with other markers such as age, female sex, fibrinogen and/or CRP (Table 1). However, when D-dimers are combined with other markers or clinical features, the sensitivity is not enhanced; in fact, it is diminished. Similarly, other markers such as fibrinogen and CRP have not demonstrated superior sensitivity. Scores from multiple diagnostic markers have been only evaluated in single studies and don’t seem superior to a high D-dimer cut-off. Although the described scores, features and markers that were included in this review varied in sensitivity and specificity, they all had very high negative predictive values. Consequently, the absence of these features can at least indicate which patients do not require malignancy screening, thus avoiding unnecessary, costly or even potentially harmful diagnostics (Figure 2).

If the clinician makes the decision to screen a cryptogenic ischemic stroke patient with high suspect for occult malignancy or also an ischemic stroke with an apparent determined etiology but with high specific malignancy markers, the screening should be guided by the international recommendations for each cancer type. The proposed flowchart offers several advantages over existing scores, primarily due to its simplicity, comprehensiveness, and clinical practicality. Furthermore, it provides a general risk stratification and guidance on how to proceed with the results. The diagnostic timing may depend upon the risk stratification (high vs moderate suspicion). We suggest performing a diagnostic workup during the initial etiology assessment in patients with high suspicion of occult malignancy (see Figure 2). A malignancy screening in these patients could include a low-dose lung CT to screen for lung cancer, a FOB test to screen for colorectal cancer, and a gynecological examination or PSA measurement to screen for breast and other common gynecological cancer types or prostate cancer according to the biological sex of the patient. If one of these test results is positive further diagnostic is required. If a body CT is chosen for logistic reasons as primary screening method in patients with high suspicion of cancer related ischemic stroke, at least a FOB test should be included for screening of colorectal cancer due to the insufficient sensitivity of body CT to detect this most prevalent type of cancer among malignancy associated ischemic strokes.^1^ If the initial screening results inconclusive in the first diagnostic assessment but high predictive factors for malignancy are present, a regular follow-up in the outpatient setting should be guaranteed and should comprise all tests to cover the most prevalent cancer types which include gastrointestinal, lung, prostate, and breast cancer as recommended by respective guidelines, especially in the first year after index stroke. In the case of moderate suspicion and intermediate risk, a timely screening diagnostic in the outpatient setting should be recommended, but an immediate assessment seems to be not necessary. Cryptogenic ischemic stroke patients without markers for occult malignancy do not require a malignancy screening and other etiologies such as atrial fibrillation should be repeatedly assessed in the outpatient setting, especially in patients >75 years in whom the risk for atrial fibrillation is higher.^47^

Given the lack of prospective studies in this area, it remains unclear whether the early detection of malignancy in patients with ischemic stroke has an impact on the prognosis or mortality of the disease. The potential benefits and limitations of cancer screening in cryptogenic ischemic stroke patients are complex. Therefore, a careful risk-benefit assessment is essential when considering cancer screening in this population. Early cancer detection can significantly impact prognosis, treatment, and secondary stroke prevention. The cancer diagnosis may assist clinicians in adjusting secondary prophylaxis, such as to an oral anticoagulant, which may prevent recurrent ischemic strokes in these patients.^48^ The evidence regarding secondary prevention in ischemic stroke patients with malignancy however is beyond the scope of this review.

Fifteen retrospective studies showed the diagnostic interval between ischemic stroke and cancer diagnoses to be 1–8 months with observation periods of 2 years or less.^45^ Similar to other thromboembolic events like idiopathic deep vein thrombosis or acute myocardial infarction, where an interval of 6–12 months between event and cancer diagnosis has been described.^5,49^ Most of the studies that looked for predictive factors for malignancy are retrospective without using standardized screening techniques for the most prevalent cancer types in ischemic stroke patients. Therefore, the real prevalence of malignancy among cryptogenic ischemic stroke could be even higher. The variability of inclusion criteria and methods of data ascertainment across the studies can significantly impact the comparability of research findings. Different studies employed diverse criteria for patient selection, including cryptogenic ischemic stroke patients, stroke patients of all etiologies, or patients with known malignancy. This can lead to differences in the characteristics of the study populations, potentially affecting the observed outcomes. More prospective studies in ischemic stroke patients with standardized diagnostic workup for malignancy are needed to support the current evidence.

## Strengths and limitations

This systematic review synthesizes data from multiple studies and highlights the importance of malignancy screening in cryptogenic ischemic stroke. This review is limited by several factors, primarily the inclusion of retrospective studies. The recommendations are based on the evidence found, and the diagnostic markers described were not compared at the same time within the same group of patients. In studies where D-dimers were assessed, some authors did not specify when the measurement was conducted, for example, before/after thrombolysis/on admission. This lack of standardization could potentially result in bias in the reported D-dimer values. Additionally, the described scores have only been studied in a single study each and have not been reproduced by other authors. Nevertheless, this review aims to provide an objective overview of the available data on this topic, with the intention of informing decision-making processes.

## Conclusions

Malignancy screening in cryptogenic ischemic stroke patients can be guided by markers like D-dimers cut-offs. Diagnostic workup should focus on prevalent cancer types associated with ischemic stroke, and clinical decision-making should be informed and guided by anamnestic and clinical factors as well as by the current cancer guidelines recommendations. Prospective studies in ischemic stroke patients with standardized diagnostic workup for malignancy are needed to validate these findings and optimize screening strategies.

## Supplemental Material

sj-jpeg-1-eso-10.1177_23969873241310760 – Supplemental material for Screening tools for malignancy in patients with cryptogenic stroke: Systematic review Supplemental material, sj-jpeg-1-eso-10.1177_23969873241310760 for Screening tools for malignancy in patients with cryptogenic stroke: Systematic review by Maria P Tieck, Constanze Single, Sven Poli, Markus C Kowarik, Ulf Ziemann, Annerose Mengel and Katharina Feil in European Stroke Journal
