# Supplementary figures and images for: Screening tools for malignancy in patients with cryptogenic stroke: Systematic review
Source: Eur Stroke J. 2025 Feb 26;10(3):665–74. doi: 10.1177/23969873241310760 (PMC11866335; doi:10.1177/23969873241310760)

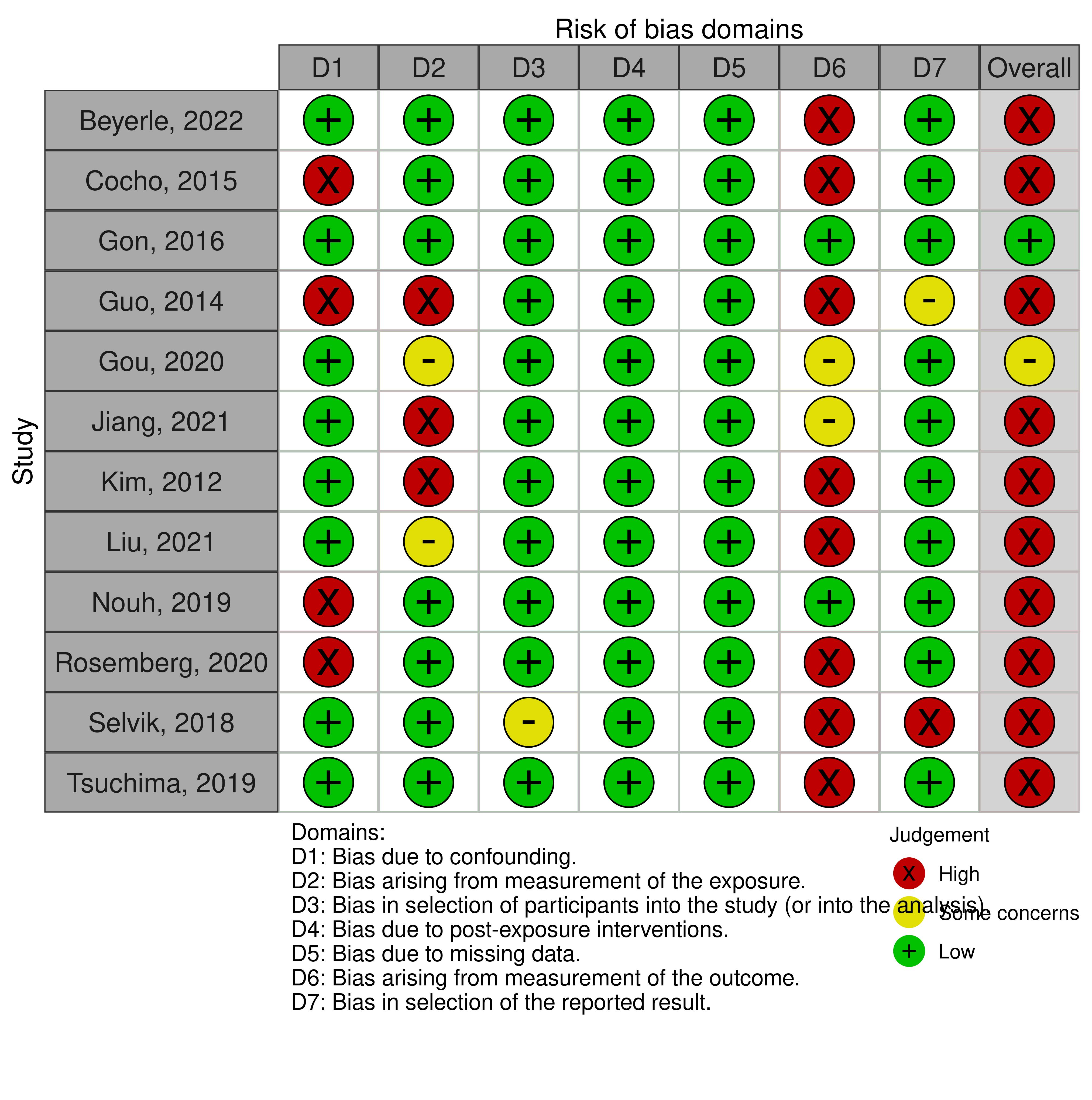

Supplement: sj-jpeg-1-eso-10.1177_23969873241310760 – Supplemental material for Screening tools for malignancy in patients with cryptogenic stroke: Systematic review [file sj-jpeg-1-eso-10.1177_23969873241310760.jpeg]
